# Improving Adherence to Infection Prevention and Disinfection Protocols Within the Ophthalmology Department: A Two-Cycle Quality Improvement Project (QIP)

**DOI:** 10.7759/cureus.109333

**Published:** 2026-05-21

**Authors:** Mashair Bakheet, Attiqa Chaudhary, Ghazai A Al Thubaiti, Abdullah A Al Assiri

**Affiliations:** 1 Ophthalmology, Magrabi Health, Riyadh, SAU; 2 Medicine, Austin Health, Melbourne, AUS

**Keywords:** environmental cleaning, hand hygeine, infection control, ophthalmology, personal protective equipment (ppe), plan-do-study-act (pdsa), quality improvement, quality improvement project (qip), staff training

## Abstract

This study evaluated and enhanced infection control practices in ophthalmology clinics at Magrabi Hospital, Riyadh, through a two-cycle quality improvement project (QIP). In the first cycle, a structured questionnaire assessed staff awareness, training, and implementation of infection prevention protocols, including hand hygiene, personal protective equipment (PPE) use, instrument disinfection, and environmental cleaning. Based on the identified gaps, targeted interventions, such as revised guidelines and online training, were introduced. The second cycle used a follow-up survey to reassess compliance and effectiveness. Results showed significant improvements in protocol awareness, hand hygiene, PPE use, disinfection log maintenance, and a reduction in procedure-related infections. However, challenges persisted, particularly with the exclusive use of multidose eye drops and unclear cleaning responsibilities. The study underscores the need for ongoing education, policy updates, and clear role definitions to maintain high infection control standards in ophthalmic settings.

## Introduction

In ophthalmology, there are various ways that can lead to healthcare-related infections. Transmission could occur through instruments or equipment that are used multiple times across different patients, increasing the potential risk of infection transmission between patients, especially for viruses and bacteria. Some studies have demonstrated that transmission of hepatitis B virus, hepatitis C virus, human immunodeficiency virus (HIV), and Creutzfeldt-Jakob disease can occur between patients during the use of tonometers for glaucoma monitoring [1,2].

Infection can also be transmitted through healthcare workers if they do not follow standard procedures or precautions to reduce the risk of infection, such as hand hygiene or the use of personal protective equipment (PPE) [3]. In 2017, in Los Angeles, an outbreak of epidemic keratoconjunctivitis secondary to adenovirus was linked to a single optometry clinic [4]. Similarly, suboptimal infection prevention practices were identified during an outbreak of epidemic keratoconjunctivitis in an eye clinic in British Columbia [5]. Thus, infection control protocols and their implementation in ophthalmology settings are important for optimal clinical practice, not only to protect patients but also to keep staff safe from infection [3].

Respiratory tract infections can be transmitted more rapidly in an ophthalmic setting because healthcare workers operate in very close proximity to patients during examinations or procedures and are therefore at risk of acquiring and transmitting these infections [3].

Infection prevention and control (IPC) is an important aspect of the healthcare system that helps implement policies and procedures to reduce the risk of infection to patients attending a healthcare facility [3,6]. It is equally important to determine whether healthcare workers have appropriate training and can implement these IPC policies. Quality improvement projects (QIPs) are an efficient way to assess the effectiveness of infection prevention protocols (IPPs) and identify areas for improvement [7].

We therefore sought to assess awareness and implementation of infection control policies at Magrabi Hospital, Riyadh, a multispecialty ophthalmology clinic. The primary objective of this study was to determine current infection control practices, with a secondary aim of identifying knowledge and practice gaps and ultimately strengthening infection prevention protocols.

## Materials and methods

This QIP was conducted at Magrabi Hospital in Riyadh, Saudi Arabia. The study was conducted in line with institutional clinical governance guidelines, and there was no requirement to obtain ethical approval.

Participants

All healthcare providers directly involved in patient care (ophthalmologists, optometrists, and ophthalmic nurses) in the ophthalmology clinics were eligible and invited to participate in the survey. Those who were on leave were excluded. Responses were collected pre- and post-intervention using a questionnaire (see the Appendices). All responses were included in both cycles 1 and 2.

QIP cycles

This QIP was conducted in two distinct cycles from July 2025 to December 2025. The infection control standards of the hospital are in accordance with MOH (Ministry of Health) and CBAHI (Saudi Central Board for Accreditation of Healthcare Institutions) frameworks, requiring a comprehensive, risk-based program that includes standard precautions, environmental hygiene, surveillance of healthcare-associated infections, staff training, and continuous monitoring to ensure patient and staff safety. A self-administered questionnaire with three main components (infection protocol awareness and training, safe practices and personal protective equipment use, and cleaning and disinfection practices) was used (Appendices). A two-cycle Plan-Do-Study-Act (PDSA) methodology was used throughout this QIP to assess and improve the infection control protocol [8]. The objectives of this QIP were Specific, Measurable, Assignable, Realistic, and Timebound (SMART) [9] and were led by the investigating doctors with the support of the quality improvement and infection control team.

A baseline assessment was performed using a questionnaire (Appendices) during cycle 1 (July 2025) to identify the objectives of the QIP. Areas for improvement were identified in cycle 1, and targeted interventions were implemented, including revised guidelines and online training for all healthcare staff.

A three-month interval between PDSA cycles was selected to allow sufficient time for implementation, adaptation, and stabilization of disinfection practices. Although rapid cycles are encouraged, there is no fixed duration in PDSA methodology, and healthcare studies report cycles lasting several months. In infection prevention, longer intervals are often necessary to generate reliable longitudinal data and distinguish sustained improvement from short-term variation. This approach supports robust evaluation while aligning with continuous improvement principles advocated by organizations such as the World Health Organization and Centers for Disease Control and Prevention.

After three months, reassessment was performed during cycle 2 (November 2025) using an identical audit tool, which was a questionnaire (Appendices). Participants in cycles 1 and 2 were drawn from the same defined clinical population and represented the same cohort of staff within the unit.

Additional areas for improvement were identified in cycle 2, and a structured training program has been planned on a regular basis for sustained quality improvement and periodic reassessment.

PDSA cycle 1: baseline assessment

The objectives and measures of this QIP were identified in cycle 1. A structured questionnaire was administered to clinical staff members, including ophthalmologists, optometrists, and nurses, at Magrabi Hospital, Riyadh. The questionnaire assessed current practices in key infection control areas, including IPP awareness and training, safe practices and PPE use, and disinfection and cleaning of medical instruments (e.g., tonometer) and clinical surfaces. The aim was to identify areas where infection control measures were insufficient or inconsistently applied.

PDSA cycle 2: intervention and reassessment

Based on the findings from the initial survey, targeted improvements were made to the existing infection control protocol.

Based on findings from cycle 1 of the QIP, guidelines were improved, including visual guides for infection protocols and hand hygiene, emphasis on mask use, and increased awareness regarding maintenance of logs and reminders from quality control teams to ensure regular training. All clinical staff were provided with online training, and it was ensured that new staff would receive training at the time of induction, with regular annual training sessions conducted thereafter.

After three months, a follow-up survey was conducted to assess the effectiveness of the implemented changes and to evaluate improvements in infection control practices. Progress, barriers, and unexpected observations were documented throughout the project.

Statistical analysis

Data were organized and analyzed using Microsoft Excel (Microsoft® Excel® for Microsoft 365 MSO, Version 2601 Build 16.0.19628.20132, 64-bit, Microsoft Corporation, Redmond, Washington), and tests of statistical significance were performed using Fisher's exact test for non-Gaussian data. The questionnaire responses consisted of binary categorical variables (e.g., yes/no compliance items), making analysis of proportions appropriate. Fisher’s exact test was selected because the study involved small sample sizes and several cells with low expected frequencies, for which it provides a robust method for analyzing categorical data without reliance on large-sample assumptions. Results were presented as absolute numbers (n) and percentages (%).

## Results

The overall summary of the results obtained in cycle 1 is presented in Table 1. A complete evaluation of the interventions was carried out, and findings were compared, as shown in Table 2. Outcome and balance measures of the PDSA were arranged in Table 3 and Table 4, respectively.

Cycle 1

**Figure 1:**
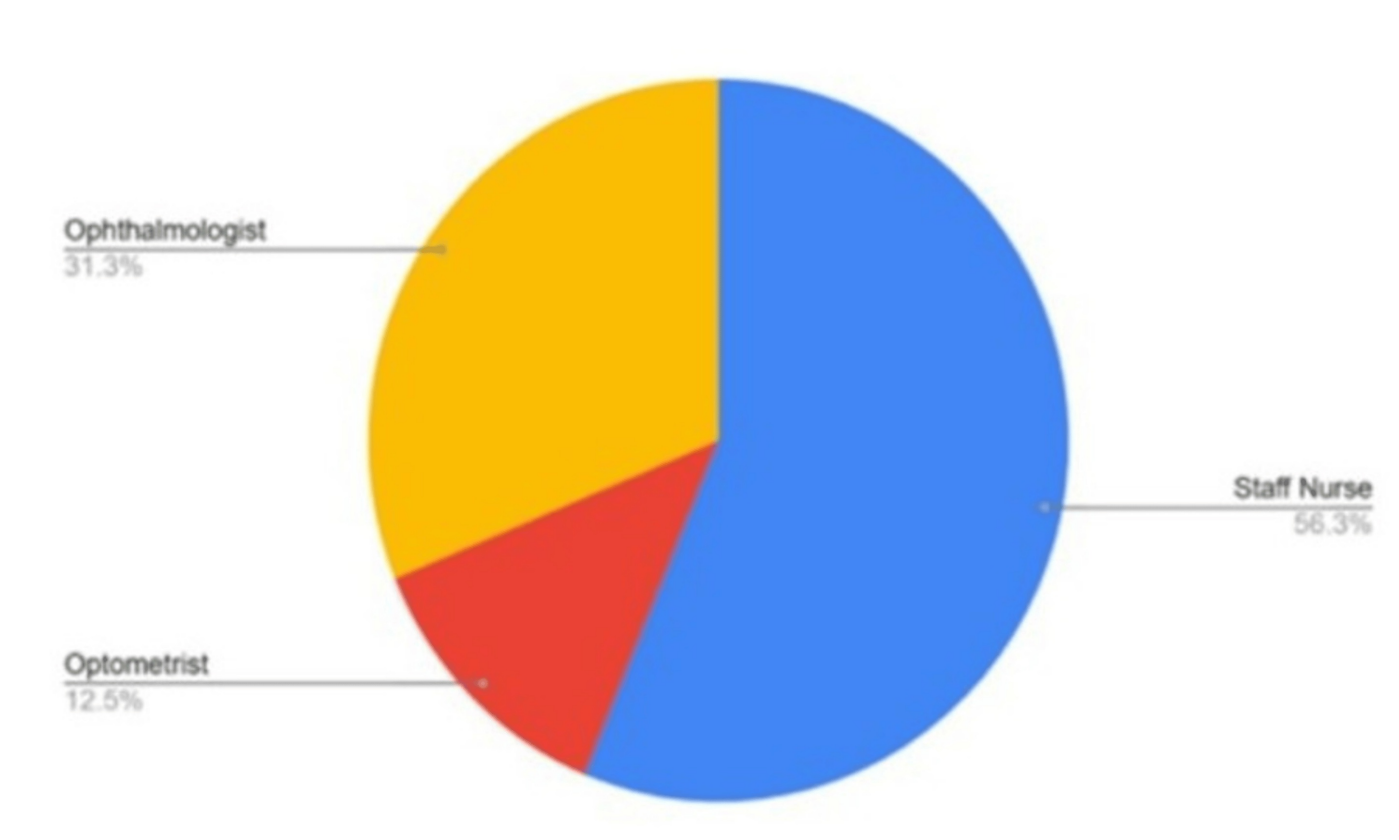
Distribution of staff members in cycle 1 of QIP Note: Total responses (n = 48): ophthalmologists (n = 15, 31.3%), nurses (n = 27, 56.3%), and optometrists (n = 6, 12.5%).

The survey was conducted at Magrabi Hospital, Riyadh, following discussion and approval from the infection control committee. A total of 48 staff members participated during the first cycle of the QIP, comprising ophthalmologists (n = 15, 31.3%), nursing staff (n = 27, 56.3%), and optometrists (n = 6, 12.5%), as shown in Figure 1.

Table 1 summarizes staff participation and baseline compliance with infection prevention and control practices among clinic staff (n = 48).

**Table 1:** Consolidated responses derived from questionnaires from cycle 1 of QIP. Data are presented as total participants (n), number of respondents (n), and corresponding percentages (%) for each indicator across predefined domains. Black bins are used for disposal of non-clinical and non-infected waste. PPE: personal protective equipment, QIP: quality improvement project.

Category	Indicator	Number of selected responses (n)	Total number of participants (n = 48)	Percentage (%)
Staff participation	Ophthalmologists	15	48	31.3
	Nursing staff	27	48	56.3
	Optometrists	6	48	12.5
Protocol awareness & training	Aware of disinfection policy	42	48	87.5
	Protocol reviewed annually	27	48	56.3
	Staff trained on protocol	42	48	87.5
	Infection control officer present	45	48	93.8
	Annual training conducted	22	48	45.8
	Procedures displayed at workstations	35	48	72.9
Hand hygiene	Use of soap and water	47	48	97.9
	Wear gloves when examining	33	48	68.8
	Hand hygiene audited	39	48	81.3
Waste disposal	Use of a black bin for non-infected PPE	39	48	81.3
Multidose eye drops	Labelled with opening date	42	48	87.5
	Discarded after 28 days	38	48	79.2
	Used for only one patient	28	48	58.3
	Tip contact with eye avoided	46	48	95.8
Respiratory prevention	Mask, breath shield use	37	48	77.1
	Slit lamp shields cleaned regularly	27	48	56.3
	Staff required to wear masks	19	48	39.6
environmental cleaning	High-touch surfaces cleaned after each patient	32	48	66.7
	Use of approved disinfectants	36	48	75.0
	Waiting/reception cleaned regularly	42	48	87.5
	Shared responsibility (staff + cleaners)	36	48	75.0
Instrument disinfection	Use of Minutes® wipes	36	48	75.0
	Use of disposable tonometer tips	3	48	6.3
	Tonometer tips disinfected after each patient	46	48	95.8
	Diagnostic lenses disinfected after each patient	33	48	68.8
	Wipe instrument with 70% isopropyl alcohol and air dry	36	48	75.0
Compliance & reporting	Disinfection logs maintained	36	48	75.0
	Infections linked to clinic procedures (reported)	37	48	77.1

Infection Protocol Awareness and Training

Most respondents (87.5%, n = 42/48) were aware of the disinfection policy and confirmed that they had received training on the disinfection protocol. However, only 56.3% (n = 27/48) reported an annual review of the policy. Out of 48 participants, 22 (45.8%) reported attending annual training sessions. Regarding disinfection procedures displayed at the workstation, 72.9% (n = 35/48) were aware of them. Regarding compliance and incident reporting, 77.1% (n = 37/48) documented and reported incidents related to clinical procedures. Procedure-related infection was defined as an infection occurring during or after a healthcare procedure that was not present or incubating before the intervention and was attributable to the healthcare exposure or procedure performed [10]. Disinfection logs were maintained by 75% (n = 36/48) of the healthcare staff.

Safe Practices and PPE Use

Hand hygiene practice was consistently followed by the majority, as 47/48 participants (97.9%) reported using soap and water for hand hygiene, and 81.3% confirmed regular audits of hand hygiene practices. However, only 68.8% (n = 33/48) reported wearing gloves during examinations. Use of preventive measures in cases presenting with respiratory tract infection, such as masks and breath shields, was reported by 77.1% (n = 37/48) of participants, while regular cleaning of slit-lamp shields was reported by 56.3% (n = 27/48). Regular use of face masks among staff was low at 39.6% (n = 19/48).

Labeling of multidose eye drops with the opening date was reported by most staff (87.5%, n = 42/48), but 79.2% (n = 38/48) reported discarding them after 28 days. However, 58.3% (n = 28/48) reported restricting the use of multidose drops to a single patient. Forty-six out of 48 (95.8%) reported that eye contact with the bottle tip was avoided. Disposal of non-infected personal protective equipment (PPE) in black bins was reported by 81.3% (n = 39/48) of staff, indicating adherence to established protocols in this area.

Cleaning and Disinfection Practices

Instrument disinfection after each patient, such as tonometer tips and diagnostic lenses, was reported by 95.8% (n = 46/48) and 68.8% (n = 33/48) of staff, respectively. Whereas 6.3% (n = 3/48) of staff reported use of a disposable tonometer. The most common method for cleaning instruments was 70% isopropyl alcohol (75.0%, n = 36/48) and minute wipes (75.0%, n = 36/48). Regarding environmental disinfection, 75.0% (n = 36/48) reported it as a shared responsibility between healthcare workers and cleaning personnel. Routine cleaning of waiting and reception areas was reported by 87.5% (n = 42/48), with approved disinfectant use reported by 75.0% (n = 36/48). Two-thirds (66.7%, n = 32/48) of staff confirmed cleaning of high-touch surfaces after each patient.

Cycle 2

**Figure 2:**
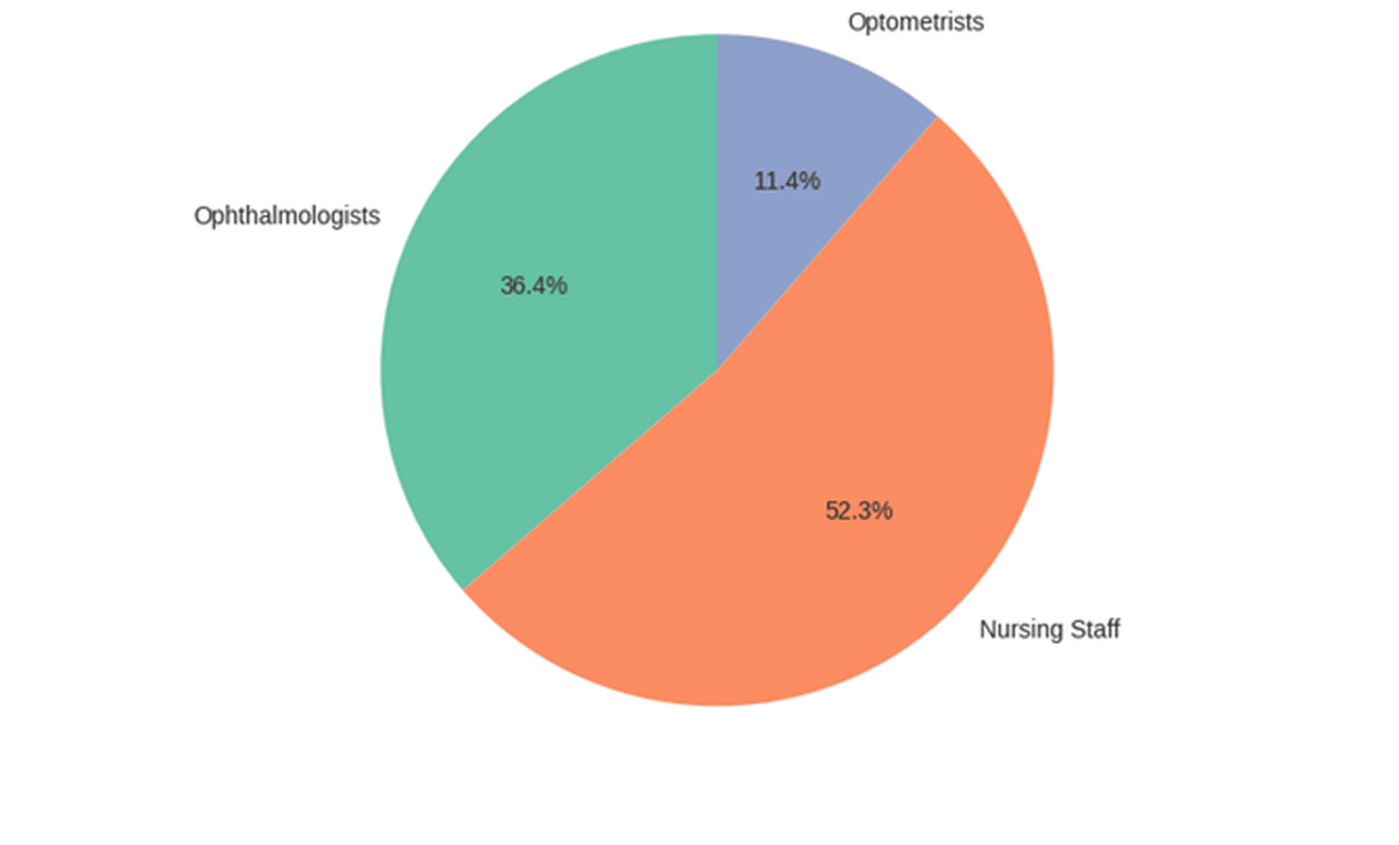
Distribution of staff members in cycle 2 of QIP Note: Total responses (n = 44): ophthalmologists (n = 16, 36.4%), nurses (n = 23, 52.3%), and optometrists (n = 5, 11.4%). QIP: quality improvement project.

The infection control team prepared a revised training module based on the recommendations from cycle 1 of the QIP. There were 44 staff members who participated in the second phase of QIP after receiving an online training session from the quality control team at Magrabi Hospital. Sixteen ophthalmologists, twenty-three nurses, and five optometrists participated in the second cycle of the QIP. The distribution of staff members in cycle 2 was shown in Figure 2. 

Improvements were observed across most of the infection control domains in cycle 2. Table 2 summarizes the comparison of infection prevention and control compliance indicators between cycle 1 and cycle 2 across all domains. Data was arranged as percentages N (%), percentage calculated as cycle 2 minus cycle 1, and p-values (Fisher’s exact test).

**Table 2:** Comparison of compliance indicators between cycle 1 (baseline) and cycle 2 (intervention and reassessment) across key domains Note: Data are presented as N (%), with percentage change calculated as cycle 2 minus cycle 1. P-values derived using Fisher’s exact test; statistically significant results are marked with an asterisk (*). PPE: personal protective equipment.

Domain	Indicator	Cycle 1 (%)	Cycle 2 (%)	% Change	p value
Protocol & training	Aware of disinfection policy	87.5	93.2	+5.7	0.49
	Protocol reviewed annually	56.3	61.4	+5.1	0.68
	Staff trained on protocol	87.5	88.6	+1.1	1.00
	Infection control officer present	93.8	88.6	−5.2	0.47
	Annual training conducted	45.8	63.4	+17.6	0.10
	Procedures displayed	72.9	86.4	+13.5	0.13
Hand hygiene	Use of soap and water	97.9	100	+2.1	1.00
	Wear gloves when examining	68.8	72.7	+3.9	0.82
	Hand hygiene audited	81.3	88.6	+7.3	0.39
Waste disposal	Black bin for non-infected PPE	81.3	90.9	+9.6	0.24
Multidose eye drops	Labelled with opening date	87.5	86.4	−1.1	1.00
	Discarded after 28 days	79.2	81.8	+2.6	0.80
	Used for only one patient	58.3	18.2	−40.1	<0.01*
	Tip contact with eye avoided	95.8	97.7	+1.9	1.00
Respiratory prevention	Mask/breath shield use	77.1	84.1	+7.0	0.37
	Slit lamp shields cleaned	56.3	72.7	+16.4	0.13
	Staff required to wear masks	39.6	68.2	+28.6	0.01*
Environmental cleaning	High-touch surfaces cleaned	66.7	72.7	+6.0	0.65
	Approved disinfectants used	75.0	88.6	+13.6	0.13
	Waiting/reception cleaned	87.5	97.7	+10.2	0.24
	Shared responsibility	75.0	54.5	−20.5	0.18
Instrument disinfection	Minutes® wipes used	75.0	79.5	+4.5	0.24
	Disposable tonometer tips	6.3	6.8	+0.5	1.00
	Tonometer tips disinfected	95.8	97.7	+1.9	1.00
	Diagnostic lenses disinfected	68.8	68.2	−0.6	0.82
	Wipe with 70% alcohol & air dry	75.0	79.5	+4.5	0.24
Compliance & reporting	Disinfection logs maintained	75.0	95.4	+20.4	0.01*
	Recent infections reported	77.1	25.0	−52.1	<0.01*

Infection Protocol Awareness and Training

During the second cycle of the QIP, significant improvements were observed across several key areas of infection control. Awareness of the disinfection policy increased from 87.5% to 93.2%, reflecting improved understanding among staff. Training on the protocol remained consistently high at 88.6%, while participation in annual training sessions rose from 45.8% to 63.4%. In addition, 86.4% of staff reported appropriate display of infection control procedures at the workstations, a rise of 13.5%. However, it is worth noting that in cycle 2, only 88.6% of staff were aware of the infection control officer’s presence, compared to 93.8% in cycle 1. Compliance and incident reporting also showed a marked improvement during the second cycle of this QIP. The proportion of staff maintaining disinfection logs improved from 75% to 95.4%; similarly, there was a significant drop in procedure-related infections from 77.1% to 25%.

Safe Practices and PPE Use

During cycle 2, hand hygiene compliance increased from 97.9% to 100%, and a similar trend was observed in glove use, which increased from 68.8% to 72.7%. Also, 88.6% of staff, compared to 81.3% in the first cycle, reported regular audits of hand hygiene practices, reflecting enhanced monitoring and accountability. Similarly, respiratory infection prevention measures showed significant progress. The use of masks and breath shields increased from 77.1% to 84.1%, while regular cleaning of slit-lamp shields improved from 56.3% to 72.7%. The second cycle of the QIP showed a marked improvement in mask wear from 39.6% to 68.2%. Labeling of eye drops in cycle 2 declined slightly from 87.5% to 86.4%; however, discarding the drops after the expiry date rose from 79.2% to 81.8%. The area of major concern was a significant drop in the use of single-use multidose eye drops from 58.3% to 18.2%, which might pose a potential risk of spreading infection, although hospital policies, in accordance with clinical guidelines in Saudi Arabia (endorsed by the Ministry of Health), mandate that opened multidose containers of eye drops should be discarded four weeks (28 days) after opening, unless the manufacturer specifies a different duration. Compliance with waste disposal (non-infected PPE) also improved from 81% to 90.9%.

Cleaning and Disinfection Practices

There was consistently high compliance with disinfection of instruments, for tonometer tips (95.8% to 97.7%) and diagnostic lenses that make contact with the eye during examination and procedures, such as gonioscopy and fundus examination lenses (Goldmann three-mirror lens and Mainster wide-field lens), which need to be cleaned for each patient, remained almost the same (68.8% to 68.2%). However, there was a slight improvement in the use of both alcohol-based cleaning methods and minute wipes from 75.0% to 79.5%. The use of disposable tonometer tips remained low at 6.8% in cycle 2. Environmental cleaning showed overall improvement across cycles. High-touch surface cleaning, along with the use of approved disinfectants, increased from 66.7% to 72.7% and 75.0% to 88.6%, respectively. Reported cleaning of waiting areas also improved significantly (87.5% to 97.7%). It is cleaned at the start of the clinic, during clinical staff shift changes, and at the end of the clinic session. However, a significant decline was observed in shared responsibility for cleaning, from 75.0% to 54.5%, indicating a need for clearer role allocation and communication between the clinical and cleaning teams.

QI measures during Cycles 1 and 2

This QIP incorporated all sets of quality improvement measures, including structure measures, process measures, outcome measures, and balancing measures.

Structure Measures

In this QIP, structure measures focused on assessing whether essential infection control infrastructure is available and adequately maintained. This included verifying the presence of a designated quality control/infection control officer, assessing the availability and accessibility of hand sanitizers at all key clinical points, and confirming the provision of clearly labeled disposal bins for infected and non-infected PPE. These elements reflected the foundational capacity of the department to support effective infection prevention behaviors.

Process Measures

**Figure 3:**
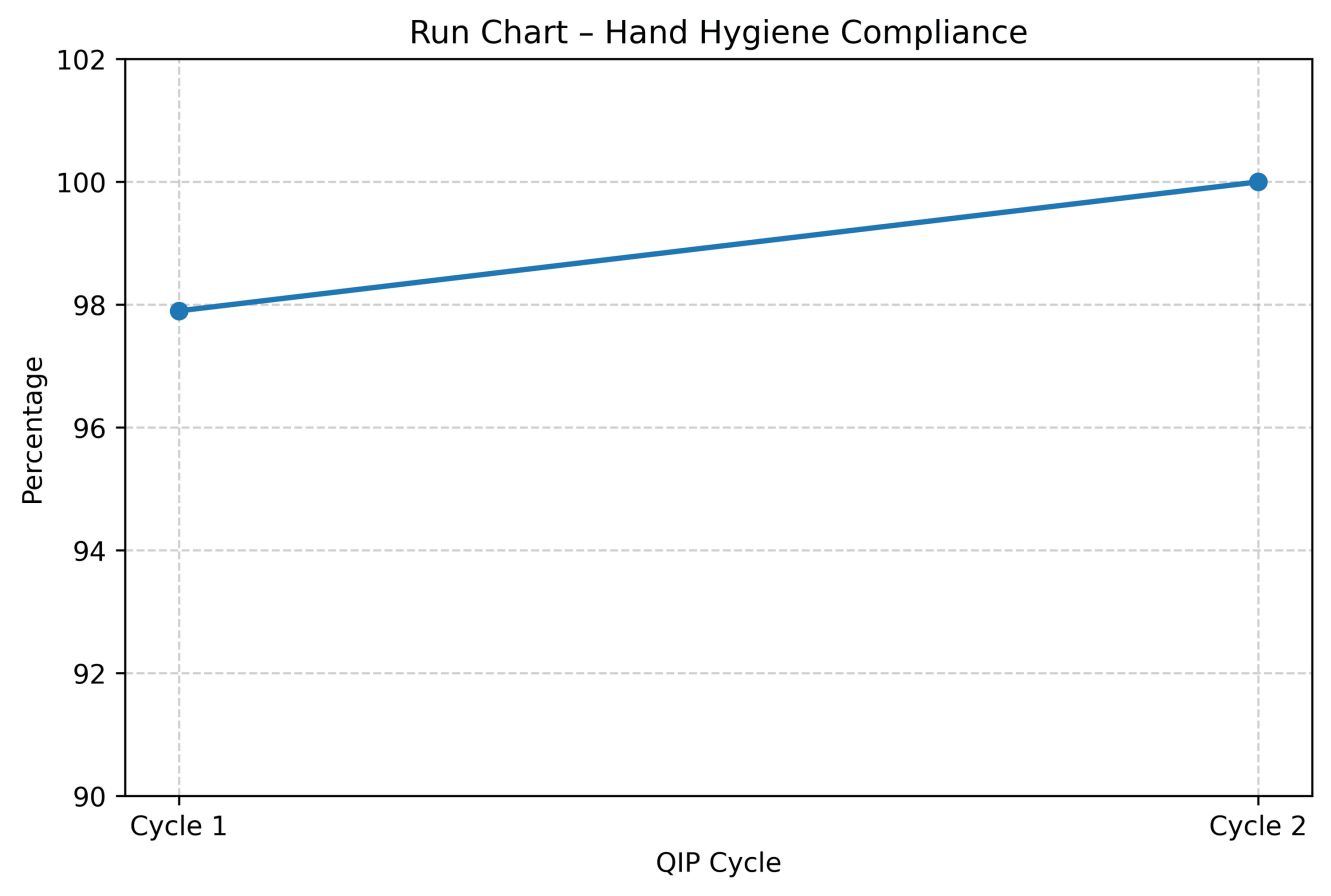
Process measures demonstrating improvement in hand hygiene compliance from 97.9% (cycle 1) to 100% (cycle 2) QIP: quality improvement project.

Process measures examined whether staff consistently followed recommended infection control procedures during routine clinical activities. For this project, process measures included assessing compliance with all steps of the disinfection protocol, evaluating adherence to proper PPE use, observing hand hygiene practices, which improved from 97.9% to 100% as shown in Figure 3, and checking staff behavior regarding the correct labeling, handling, and disposal of multidose ophthalmic drops. These indicators captured the daily implementation of infection control standards and highlighted the specific areas where practice may deviate from guidelines. 

Outcome Measures

Outcome measures evaluated the overall effectiveness of the QIP and the impact of improved practices on patient safety. The primary outcomes of this project include achieving more than 90% compliance across all infection control domains compared with baseline performance. Additionally, outcome measures aim to demonstrate a reduction in the incidence of procedure-related infections, reflecting the goal of enhancing patient safety and quality of care. The results are summarized in Table 3.

**Table 3:** Outcome measures: performance difference (percentage) between cycle 1 and cycle 2 Note: Outcome measures (patient safety practices) are presented as percentages (%) in both cycles, with performance differences expressed as percentage change (%). QIP: quality improvement project.

QIP-domains	Cycle 1 (%)	Cycle 2 (%)	Performance difference (%)
Staff required to wear masks	39.6%	68.2%	↑ +28.6%
Slit-lamp shield cleaning	56.3%	72.7%	↑ +16.4%
Hand hygiene audited	81.3%	88.6%	↑ +7.3%
Infections reported	77.1%	25.0%	↓ −52.1% (Major)
Logs maintained	75.0%	95.4%	↑ +20.4%
Waiting area cleaning	87.5%	97.7%	↑ +10.2%
High-touch surfaces cleaned	66.7%	72.7%	↑ +6.0%
Protocol awareness	87.5%	93.2%	↑ +5.7%

Balancing Measures

Balancing measures were used in cycle 2 to ensure that improvements in disinfection practices did not create unintended consequences [11]. These included monitoring the impact on clinical workflow, staff workload and documentation burden, supply availability (disposable tonometer tips), and maintenance of other infection control behaviors. A notable balancing effect was the significant decline in single-patient use of multidose eye drops (58.3% to 18.2%), suggesting reduced vigilance in this domain following increased emphasis on other areas during training [11].

**Figure 4:**
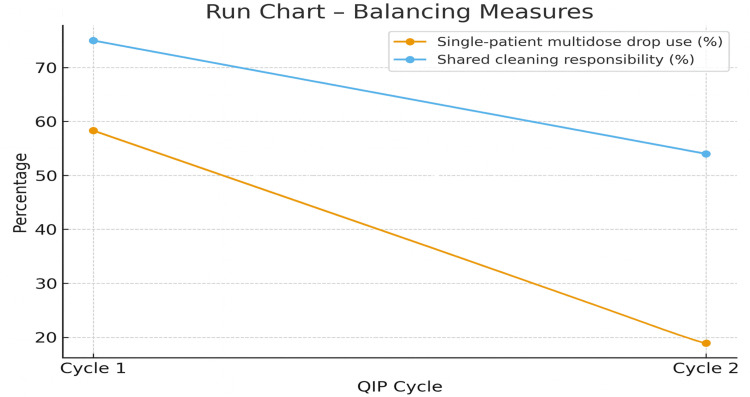
Balancing measure demonstrating trends in single-patient multidose eye drop use (%) and shared cleaning responsibility (%) across cycles 1 and 2 to assess unintended effects of the quality improvement interventions QIP: quality improvement project.

Additionally, shared cleaning responsibility decreased from 75% to 54.5%, indicating potential confusion regarding staff versus cleaning team roles, as shown in Figure 4. 

These findings, as shown in Table 4, highlighted the importance of continuous monitoring to ensure that improvements in one area do not compromise others.

**Table 4:** Balancing measures: performance difference (percentage) between cycle 1 and cycle 2 Note: Balancing measures in cycle 2 (indicating decline in certain QIP domains) are presented as percentages (%) in both cycles, with performance differences expressed as percentage change (%).

Balancing measure	Cycle 1, n (%)	Cycle 2, n (%)	% Change
Used for only one patient (eye drops)	58.3	18.2	−40.1
Shared responsibility for cleaning	(5.0	54.5	−20.5

## Discussion

This QIP reinforces policies and procedures to ensure that both healthcare workers and patients are protected from infection. QIP is also an effective way to implement infection control protocols. The primary aim of this study was to investigate whether healthcare staff have appropriate training and knowledge of infection control procedures and policies at our institute. The secondary aim was to improve areas of concern.

The primary aim of this QIP was to evaluate trends in awareness and practice following the intervention within a real-world clinical setting, rather than to demonstrate definitive effectiveness through statistically powered hypothesis testing. While some observed improvements were modest and not statistically significant, they remain operationally relevant in the context of quality improvement and helped identify areas requiring further reinforcement and ongoing interventions.

The QIP helped identify areas for improvement in the infection control protocol. In the first cycle, a considerable proportion of staff reported a lack of annual review and training provision, which improved after policy review followed by an online training session on infection control practices. It is worth noting that the majority of staff reported the presence of infection control personnel during cycle 1, reflecting awareness of structured infection prevention governance within the ophthalmology department at Magrabi Hospital, Riyadh; however, fewer staff in cycle 2 reported awareness of infection control personnel compared to cycle 1.

Prompt reporting and documentation of errors improve staff and patient safety and enable the quality assurance team to investigate and address the root causes of errors [12]. Cycle 2 of the QIP demonstrated a significant increase in maintenance of disinfection logs, which are fundamental to quality assurance and indicate enhanced awareness of documentation practices [13]. The second cycle also demonstrated improvement in aseptic technique, resulting in a marked reduction in procedure-related infections.

After receiving training, healthcare staff showed improvement in safe practices such as glove use during patient examination, and hand hygiene compliance reached 100%, which aligns with "Your Five Moments for Hand Hygiene" recommended by the World Health Organization (WHO) [14].

Aizman et al. stated that vigorous handwashing and disinfection of instruments can decrease transmission of infections, and these simple steps are effective in preventing the spread of infections among patients [15].

Similarly, there were marked improvements in other domains, such as respiratory infection prevention (PPE use, slit-lamp barrier use, and environmental cleaning), which are recommended to prevent respiratory droplet transmission [16]. This improvement was attributed to revision of the training protocol and provision of training for all healthcare workers. It also reflects that infection prevention education and training are important tools to reduce the risk of infection transmission and associated healthcare costs [17,18].

However, a significant decline in restricting multidose eye drops to single-patient use was observed, which is concerning, as repeated use of multidose eye drops has been associated with microbial contamination of dropper tips and solutions, with documented risks of cross-contamination and subsequent ocular infections [19].

Thus, further investigation and policy review are required to ensure compliance with recommended ophthalmic infection control guidelines. Clearly defining team roles and responsibilities could help improve compliance with IPP [20].

Environmental and instrumental cleaning are key elements of IPC. A study conducted by Sobolewska et al. found high bacterial contamination on slit lamps and portable devices, which could lead to cross-contamination between staff members, emphasizing the importance of proper instrument cleaning [21]. These indicators remained strong, with a positive trend seen in cycle 2 of the QIP. Isopropyl alcohol and minute wipes remained the preferred disinfectants in both cycles.

However, there were still areas noted during the second cycle of the QIP that warrant further education and monitoring. The observed reduction in awareness of the IPC officer (93.8% to 88.6%) is considered a meaningful real-world signal rather than random variation alone, suggesting a potential gap in role visibility or retention of key governance information following the intervention period. This may reflect ongoing issues such as competing clinical priorities, staff turnover, or insufficient reinforcement of institutional IPC leadership structures during training.

A very small proportion of staff reported using disposable tonometer tips, which may be due to preference for other devices or limited availability. This finding requires further monitoring to ensure quality of care is not compromised. A significant regression was also observed in shared responsibility for cleaning, which declined markedly in cycle 2. This suggests reduced clarity regarding staff roles, underscoring the need for focused education complemented by clear visual guidance, including posters and graphics [15]. Implementation of these measures may help reduce economic burden and mitigate infection risk [22]. This study demonstrates that sustained education, targeted interventions, and refresher training are important tools for achieving compliance with international infection control standards [23]. It is also important to create a culture of shared responsibility, which encourages staff to learn from one another and actively participate in infection prevention measures, thereby reducing the risk of cross-infection [12].

The strengths of this project include a focused clinical question, use of a structured PDSA methodology across two cycles, and improvement in most domains assessed under this QIP.

Limitations

The limitations of this study were variability in sample size between audit cycles, reliance on self-reported practices, and potential observer bias. Procedure-related infections were based on self-reported practices rather than objectively confirmed infection surveillance data. Therefore, the observed decline may reflect changes in reporting behavior, awareness, or recall bias in addition to potential improvement in infection control practices. The manuscript has been revised to clarify that this finding represents an observed trend within the QIP context and should not be interpreted as definitive evidence of reduced infection incidence

As the project primarily assessed reported awareness and practices rather than directly observed behaviors, the findings may not fully reflect actual day-to-day clinical practice. This limitation should therefore be considered when interpreting the reported improvements in compliance outcomes.

Despite these challenges, this audit showed strong evidence of organizational improvement and safety among staff members of the multi-subspecialty ophthalmology clinics of Magrabi Hospital, Riyadh.

Future directions

This study helped the infection control team at Magrabi hospital, Riyadh, to revise their guidelines to make sure that continuous education about IPC for health care workers. It also led to deciding to run QIP in the future to identify the gaps in education and improve the quality of services to the patients.

## Conclusions

This two-cycle QIP improved infection control practices among clinical staff in the ophthalmology department at Magrabi Hospital, Riyadh. Systematic assessment, targeted interventions, and follow-up led to improved hand hygiene, PPE use, disinfection procedures for the environment and instruments, and documentation compliance. The project was also able to identify the new challenges, such as the proper use of multidose eye drops and the clear assignment of cleaning responsibilities among team members. These results emphasize the need for continuous education, regular policy reviews, and effective communication with staff. Continued training and periodic assessments through various audit tools are essential to maintain high quality standards for patient and staff safety in the ophthalmology clinics.

## Figures and Tables

**Figure 5:**
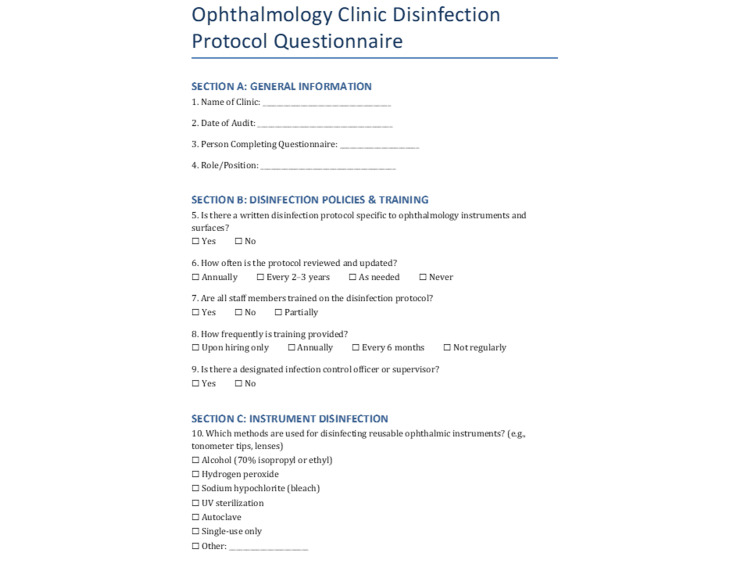
Ophthalmology clinic disinfection protocol questionnaire (Part 1)

**Figure 6:**
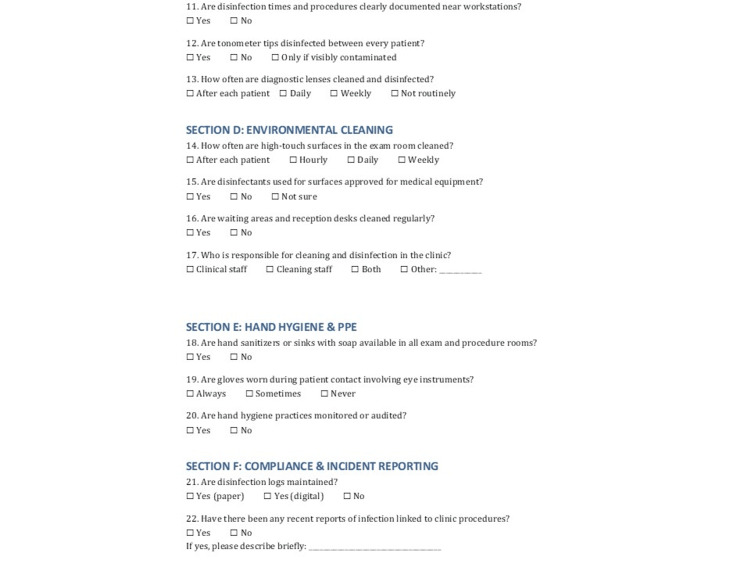
Ophthalmology clinic disinfection protocol questionnaire (Part 2)

**Figure 7:**
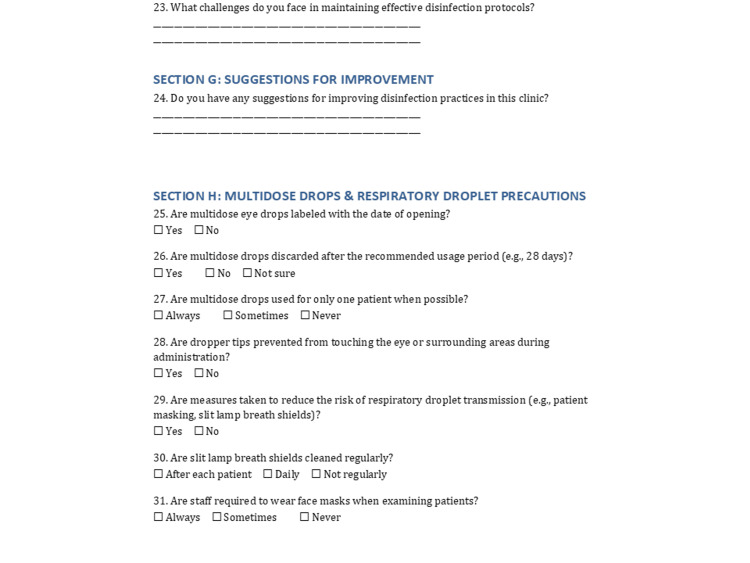
Ophthalmology clinic disinfection protocol questionnaire (Part 3)
